# Interaction with indoor plants may reduce psychological and physiological stress by suppressing autonomic nervous system activity in young adults: a randomized crossover study

**DOI:** 10.1186/s40101-015-0060-8

**Published:** 2015-04-28

**Authors:** Min-sun Lee, Juyoung Lee, Bum-Jin Park, Yoshifumi Miyazaki

**Affiliations:** Department of Horticulture Sciences, College of Agriculture and Life Sciences, Chungnam National University, 99 Daehak-ro, Yuseong-gu, Daejeon 305-764 Korea; Korea Forest Service, Government Complex 1, 189 Cheongsa-ro, Seo-gu, Daejeon 302-701 Korea; College of Agriculture and Life Sciences, Chungnam National University, 99 Daehak-ro, Yuseonggu, Daejeon 305-764 Korea; Center for Environment, Health and Field Sciences, Chiba University, 6-2-1 Kashiwanoha, Kashiwa, Chiba 277-0882 Japan

**Keywords:** Indoor plant, Technostress, Psychological and physiological effects, Heart rate variability, Sympathetic nervous system

## Abstract

**Background:**

Developments in information technology cause a great deal of stress to modern people, and controlling this stress now becomes an important issue. The aim of this study was to examine psychological and physiological benefits of interaction with indoor plants.

**Methods:**

The study subjects were 24 young male adults at the age of 24.9 ± 2.1 (mean ± SD). The crossover experimental design was used to compare the differences in physiological responses to a computer task and a plant-related task. Subjects were randomly distributed into two groups. The first group (12 subjects) carried out transplanting of an indoor plant, whereas the second group (12 subjects) worked on a computer task. Then, each subject switched activities. The psychological evaluation was carried out using the semantic differential method (SDM) and physiological evaluation using heart rate variability (low-frequency (LF) and high-frequency (HF) components) and blood pressure.

**Results:**

Analysis of the SDM data showed that the feelings during the transplanting task were different from that during the computer task: the subjects felt more comfortable, soothed, and natural after the transplanting task than after the computer task. The mean value of total log[LF/(LF + HF)] (sympathetic activity) increased over time during the computer task but decreased at the end of the transplanting task, and the differences were significant. Furthermore, diastolic blood pressure was significantly lower after the transplanting task.

**Conclusions:**

Our results suggest that active interaction with indoor plants can reduce physiological and psychological stress compared with mental work. This is accomplished through suppression of sympathetic nervous system activity and diastolic blood pressure and promotion of comfortable, soothed, and natural feelings.

## Background

The living space of modern people has moved from outdoors to indoors - more than 85% of a person’s daily life is spent indoors. Developments in information technology have allowed people to connect and remain connected to the computer environment. However, this diffusion of information technology causes a great deal of stress, such as technostress [1], which is a modern disease of adaptation caused by an inability to cope with the new computer technologies in a healthy manner. Many studies have been carried out to evaluate various ways to control this psychological stress; for example, the effect of a natural environment on human beings has been actively studied since the 1980s [2-4]. A number of studies are also underway concerning the physiological and psychological effect of interacting with plants. Plants relieve physiological stress and negative psychological symptoms [5-8]. This finding has important implications because the cardiovascular system can be damaged by overactivation of the sympathetic nervous system as a result of a stressful situation [9,10].

In recent years, the comforting effect of a natural environment has been verified, and further evidence-based studies are underway. Various experimental approaches have been attempted in regard to physiological measures, which can verify the beneficial effects of natural stimuli quantitatively. A contact with plants is an intuitive and nonverbal activity that can provide psychological stability and comfort by stimulating four senses in various ways. Indoor plants have drawn the attention of the scientific community because of their various benefits: they enhance job satisfaction in office workers [11], reduce psychological stress [12], improve mood states [13-16], and enhance cognitive health [17-19]. These effects can positively affect resistance to diseases and chronic stress [20,21], but rigorous evidence is lacking. With the present methods of psychological assessment, health benefits of indoor plants cannot be sufficiently explained. Furthermore, few studies have investigated the physiological mechanism underlying the health benefits due to indoor plants.

Therefore, in this study, we attempted to examine the physiological benefits of indoor plants in modern people. We focused on cardiovascular changes when a person makes a contact with foliage plants: we measured the autonomic nerve system activity. In addition, we attempted to quantify the psychological changes during the contact with plants as well.

## Methods

### Subjects and the protocol

We enrolled 24 young male adults at the age of 24.9 ± 2.1 (mean ± SD). None of the subjects reported a history of physical or psychiatric disorders. The study lasted 3 days. Alcohol and tobacco were prohibited, and caffeine intake was controlled. Prior to the start of the experiments, the subjects were fully informed of the aims and procedures of the experiments, and informed consent was obtained. This study was conducted in compliance with regulations of the Clinical Trial Center, Chungnam National University Hospital, Korea, and the Ethics Committee of the Center for Environment, Health and Field Sciences, Chiba University, Japan.

The crossover experimental design was used to compare differences in physiological responses to the two tasks. Twenty-four subjects were randomly distributed into two groups. On the first day of the experiments, the first group (12 subjects) tended to indoor plants while the second group (12 subjects) worked on a document in a word processor, one of the most typical computer tasks, which needs continuous physical activity, like the transplanting task. On the second day, the subjects switched activities. Each subject performed each task at the same time of the day to reduce the effects of diurnal variation.

## Materials

**Figure 1:**
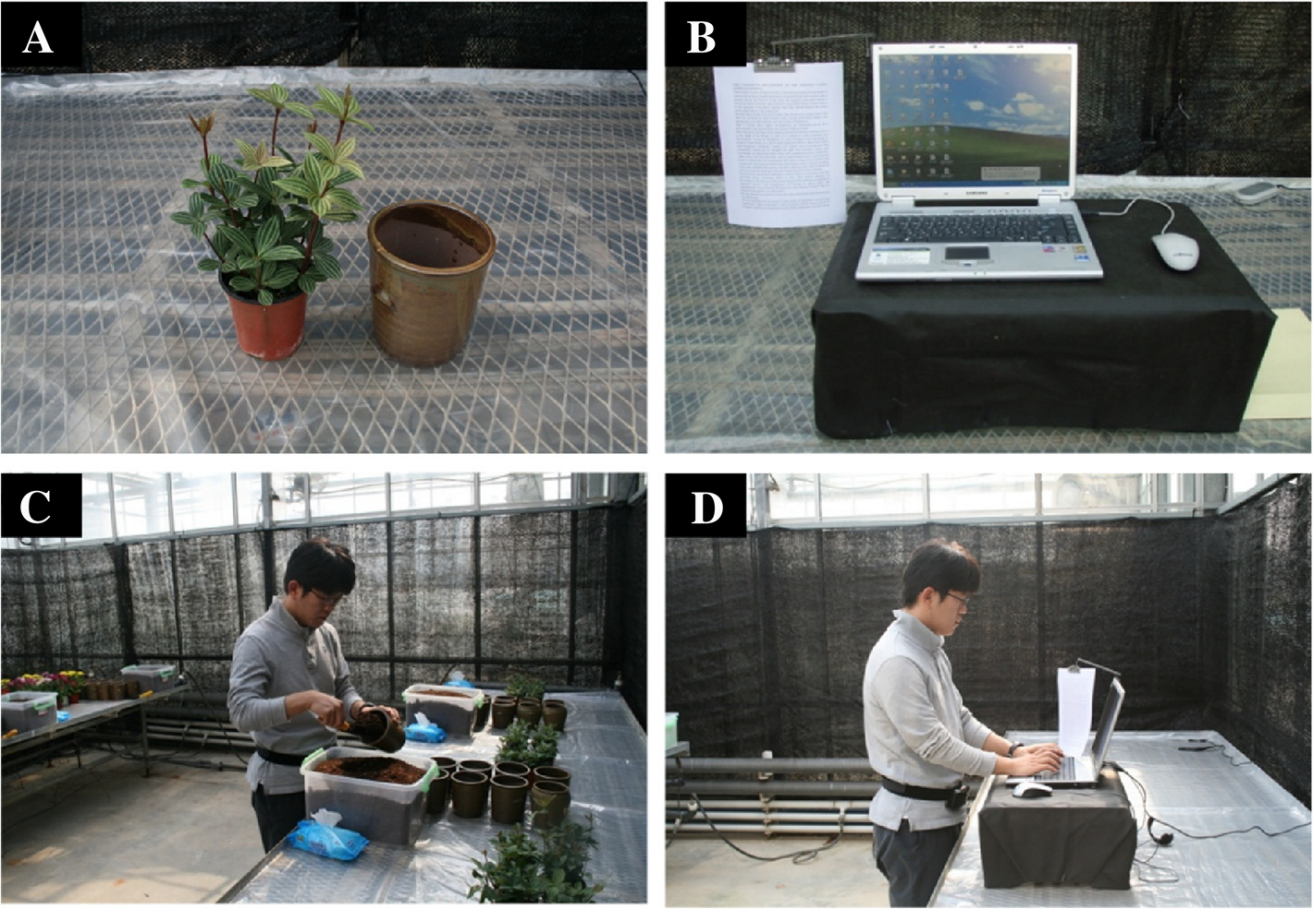
Photographs of **(A)** *Peperomia dahlstedtii*, **(B)** a computer, **(C)** a subject transplanting indoor plants, and **(D)** a subject performing a computer task.

*Peperomia dahlstedtii*, a common indoor plant, was used for the transplanting work. The transplanting method was taught to each subject prior to the experiments so that they could work more comfortably. The experiment was carried out in a greenhouse room, where the wall was covered with a black curtain (Figure 1), and environmental conditions were maintained relatively consistent (temperature 20.8°C ± 1.4°C, mean ± SD; humidity, 57.7% ± 6.6%; illuminance 1,365.5 ± 327.9 lux).

The temperature was set at 22°C, and humidity was controlled so that it would not decrease from 50%. The lighting condition was controlled at comfortable level by hanging curtains from the ceiling and on walls to protect from direct sunlight.

### Measurements

An electrode was attached to the subjects’ chest in the waiting room, and they moved to the experimental room. After a 2-min rest in a seated position, they performed the given tasks, that is, transplanting houseplants or computer work, for 15 min. Heart rate variability (HRV) was measured consecutively during the task using a portable electrocardiograph (Activtracer AC-301A; GMS, Tokyo, Japan). Blood pressure and pulse rate data were collected before and after the tasks using a digital blood pressure monitoring device (HEM-1000; OMRON, Kyoto, Japan).

### Data analysis

HRV data were calculated by averaging 1-min inter-beat (R-R) data and analyzed by means of maximum entropy methods (MemCalc, GMS, Tokyo, Japan) using the low-frequency (LF; 0.04 to 0.14 Hz) and high-frequency (HF; 0.15 to 0.40 Hz) components of the power spectrum. The HF component reflects activity of the parasympathetic nervous system, which increases in a relaxed state, and LF/(LF + HF) reflects activity of the sympathetic nervous system, which increases in a stressed state. All HRV values were log-transformed (base 10).

The feelings that the subjects experienced during the test were measured using the semantic differential method (SDM), which is a self-rating assessment. The subjects rated their feelings on a seven-point scale for three test items - ‘Comfortable’, ‘Relaxed’, and ‘Natural’ - by writing down their feelings at the moment before and after the tasks.

A paired *t* test was used to compare the differences in HRV values and blood pressure between the two tasks. Wilcoxon signed-rank test was used for the analysis of psychological data. Statistical analysis was carried out using the SPSS software, version 21.0 (IBM Corp., Chicago, IL, USA). In both cases, we applied one-sided tests because of the hypothesis that humans would feel more relaxed after the transplanting task. In all cases, the differences were considered statistically significant at *P* < 0.05.

## Results

**Figure 2:**
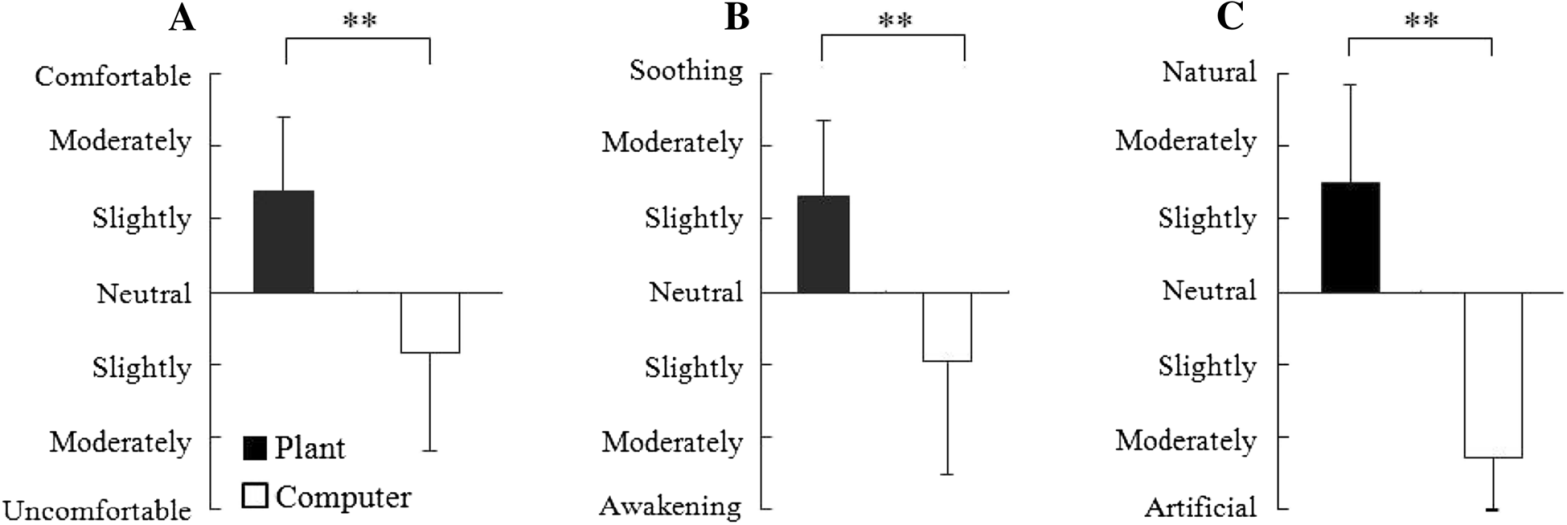
Comparison of psychological assessments between plant and computer stimuli. **(A)** Feelings of comfort, **(B)** the feeling of relaxation, and **(C)** the feeling of naturalness. *N* = 24, mean ± SD, **P < 0.01 according to the Wilcoxon signed-rank test.

Analysis of the SDM data showed that the feelings during the transplanting task were different from that during the computer task. The subjects felt comfortable, soothed, and natural after the transplanting task, whereas they felt uncomfortable, awakened, and artificial after the computer task. There were significant differences between the two for the three feelings tested after the 15-min tasks, despite the absence of significant differences in these feelings before the tasks (Figure 2) when the subjects showed generally neutral responses for these three feelings.

**Figure 3:**
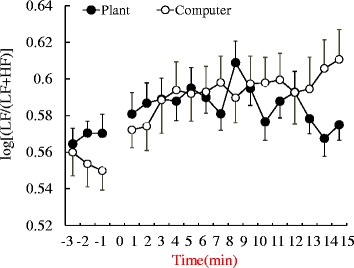
Comparison of average log[LF/(LF + HF)] of HRV during the plant and computer tasks. *N* = 24, mean ± SE. HF: high-frequency component, LF: low-frequency component.

**Figure 4:**
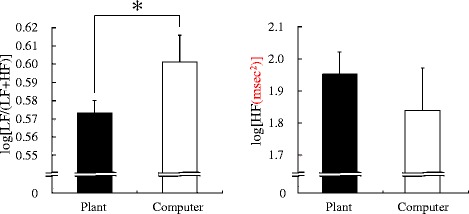
Comparison of average log[LF/(LF + HF)] and logHF of HRV during the last 3 min of plant and computer tasks. *N* = 24, mean ± SE, *P < 0.05 (paired *t* test). HF: high-frequency component, LF: low-frequency component.

**Figure 5:**
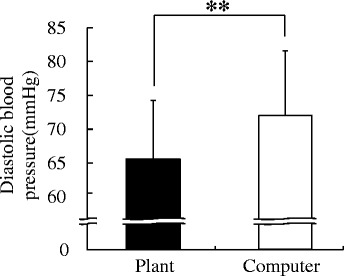
Comparison of diastolic blood pressure after the plant and computer tasks. *N* = 24, mean ± SD, **P < 0.01 (paired *t* test).

The changes of log[LF/(LF + HF)] reflecting the sympathetic nervous system activity during the tasks are shown in Figure 3. Although there were no significant differences in the mean value of total log[LF/(LF + HF)] for the 15-min period, these values changed in ways that differed between the two tasks. The log[LF/(LF + HF)] value increased over time during the computer task but decreased at the end of the transplanting task. Therefore, the data from the last 3 min were compared; they showed significant differences in log[LF/(LF + HF)] (transplanting task 0.57 ± 0.04, computer task 0.60 ± 0.05; *P* = 0.021; Figure 4). There were no significant differences in logHF values of the last 3 min between the transplanting (1.94 ± 0.12) and computer task (1.84 ± 0.12). In the analysis of diastolic blood pressure, a significant difference was observed after completion of a task (transplanting task, 65.26 ± 0.14; computer task, 71.75 ± 0.16; *P* = 0.001; Figure 5).

## Discussion

In this study, we examined the stress-reducing effects of interaction with indoor foliage plants by measuring physiological and psychological responses. The results of HRV analysis indicate that indoor plants have positive physiological effects on the autonomic nervous system by suppressing sympathetic activity, which often increases when a subject is exposed to a stressor. In this study, the value of log[LF/(LF + HF)] (corresponds to sympathetic activity) increases soon after the subjects start the tasks (either the transplanting or computer task), then shows a tendency for a slow decrease during the transplanting task, despite a consistent increase during the computer task. The stress-reducing effect was observed at the end of the transplanting task (the last 3 min); this finding may be partly consistent with that of a previous study [22]. However, this finding is on the basis of comparison with a computer task, a type of mental task, which tends to increase sympathetic nervous activity [23,24].

In the present study, the subjects were found to have positive feelings when interacting with indoor plants. In contrast, the computer task increased diastolic blood pressure and sympathetic nervous system activity. The self-rating SDM scores also indicated that working on a computer may have negative effects on the psychological state. It was assumed that participants in this study were familiar with the computer tasks in real life because we recruited university students. Nonetheless, the results showed that the subjects felt stressed when performing the computer task compared to the transplanting task; even though the latter was seemingly unfamiliar work to our subjects.

Our data support the notion that active interaction [25] with indoor plants can have positive effects on human stress response mediated by cardiovascular activities. These physiological benefits may result from multiple natural stimuli acting on the senses of vision, hearing, touch, and smell; this effect is also seen in forest therapy research [26-29]. Although many studies reported positive effects of indoor plants, most of them have been focused on the benefits of passive interaction [25] with indoor plants [11,13,30,31]. Our study presents relevant data that can explain the mechanism behind the health benefits of active interaction with indoor plants, from the standpoint of the stress response.

A possible limitation of this study is that our subject group was limited to healthy young male university students; more diverse subject groups should be tested in the future to generalize the results. In the present work, physiological responses to specific stimuli appeared at the end of the 15-min experimental period task duration; therefore, the task duration should be extended beyond 15 min in future studies. It is also recommended that future research should use a control task more realistic and practical with regard to the application of the results.

## Conclusions

Our results suggest that active interaction with indoor plants can reduce physiological and psychological stress compared with mental work. This is accomplished through suppression of sympathetic nervous system activity and diastolic blood pressure and promotion of comfortable, soothed, and natural feelings.

## Abbreviations

HF: high-frequency
HRV: heart rate variability
LF: low-frequency
SDM: semantic differential method

## References

[1] Brod C. Technostress: the human cost of the computer revolution. Reading: MA: Addison-Wesley; 1984.

[2] Ulrich RS (1984). View through a window may influence recovery from surgery. Science.

[3] Lewis CA (1996). Green nature/human nature: the meaning of plants in our lives.

[4] Kaplan R, Kaplan S (1989). The experience of nature: a psychological perspective.

[5] Chang C, Chen P (2005). Human response to window views and indoor plants in the workplace. Hort Science.

[6] Coleman CK, Mattson RH (1995). Influences of foliage plants of human stress during thermal biofeedback training. Hort Technology.

[7] Fjeld T (2000). The effect of interior planting on health and discomfort among workers and school children. Hort Technology.

[8] Moore EO (1981). A prison environment’s effect on health care service demands. J Environ Sys.

[9] Boomershine CS, Wang T, Zwilling BS, Ader R, Chone D, Felten L, Cohen N (2001). Neuroendocrine regulation of macrophage and neutrophil function. Psychoneuroimmuology.

[10] McEwen BS (1998). Protective and damaging effects of stress mediators. N Engl J Med.

[11] Dravigne A, Waliczek TM, Lineberger RD, Zajicek JM (2008). The effect of live plants and window views of green spaces on employee perceptions of job satisfaction. Hort Sci.

[12] Kaplan R (2001). The nature of the view from home: psychological benefits. Environ Behav.

[13] Adachi M, Rode CLE, Kendle AD (2000). Effects of floral and foliage displays on human emotions. Hort Sci.

[14] Ulrich RS (1981). Natural versus urban scenes: some psycho-physiological effects. Environ Behav.

[15] Ulrich RS, Simons RF, Losito BD, Fiorito E, Miles MA, Zelson M (1991). Stress recovery during exposure to natural and urban environments. J Environ Psychol.

[16] Shibata S, Suzuki N (2001). Effects of indoor foliage plants on subjects’ recovery from mental fatigue. North Am J Psychol.

[17] Cimprich B (1993). Development of an intervention to restore attention in cancer patients. Cancer Nurs.

[18] Hartig TA, Mang A, Evans GW (1991). Restorative effects of natural environment experience. Environ Behav.

[19] Tennessen CM, Cimprich B (1995). Views to nature: effects on attention. J Environ Psychol.

[20] Pearin LI, Goldberger L, Breznitz S (1993). The social contexts of stress. Handbook of stress.

[21] Salovey P, Rothman AJ, Detweiler JB, Steward WT (2000). Emotional states and physical health. Am Psychol.

[22] Lee MS, Park BJ, Lee J, Park KT, Ku JH, Lee JW (2013). Physiological relaxation induced by horticultural activity: transplanting work using flowering plants. J Physiol Anthropol.

[23] Ishibashi K, Ueda S, Yasukouchi A (1999). Effects of mental task on heart rate variability during graded head-up tilt. Appl Human Sci.

[24] Sato N, Miyake S (2004). Cardiovascular reactivity to mental stress: relationship with menstrual cycle and gender. J Physiol Anthropol Appl Human Sci.

[25] Lohr VI, Pearson-Mims CH (2005). Children’s active and passive interactions with plants influence their attitudes and action toward trees and gardening as adults. Hort Technology.

[26] Tsunetsugu Y, Park BJ, Ishii H, Hirano H, Kagawa T, Miyazaki Y (2007). Physiological effects of “Shinrin-yoku” (taking in the atmosphere of the forest) in an old-growth broadleaf forest in Yamagata prefecture, Japan. J Physiol Anthropol.

[27] Park BJ, Tsunetsugu Y, Ishii H, Furuhashi S, Hirano S, Kagawa T (2008). Physiological effects of Shinrin-yoku (taking in the atmosphere of the forest) in a mixed forest in Shinano Town, Japan. Scand J Forest Res.

[28] Lee J, Park BJ, Tsunetsugu Y, Ohira T, Kagawa T, Miyazaki Y (2011). Effect of forest bathing on physiological and psychological responses in young Japanese male subjects. Public Health.

[29] Lee J, Tsunetsugu Y, Takayama N, Park BJ, Li Q, Song CR (2014). Influence of forest therapy on cardiovascular relaxation in young adults. Evid Complement Altern Med.

[30] Lohr VI, Person-Mins CH (2000). Physical discomfort may be reduced in the presence of interior plants. Hort Technology.

[31] Bringslimark T, Hartig T, Patil GG (2009). The psychological benefits of indoor plants: a critical review of the experimental literature. J Environ Psychol.

